# Collagen Fibril Diameter in Relation to Bone Site and to Calcium/Phosphorus Ratio

**DOI:** 10.1100/tsw.2006.212

**Published:** 2006-08-31

**Authors:** Panagiotis Berillis, Dimitris Emfietzoglou, Margaret Tzaphlidou

**Affiliations:** Laboratory of Medical Physics, Medical School, Ioannina University, 45110 Ioannina, Greece

**Keywords:** fibrils, diameter, electron microscopy, cortical bone, calcium/phosphorus ratio

## Abstract

The collagen fibril diameter was measured in cortical bone samples from the femoral neck, rear and front tibia of female and male rats and rabbits using electron microscopy analysis. In most cases, statistically significant differences in mean fibril diameter values between different bone sites were detected. The order of magnitude for the above structural parameter was the same for both genders in both experimental species. In rats, the greatest mean diameter value was that for the femoral, while in rabbits, the one for the rear tibia demonstrating a dependence on bone use and life style. An important aspect was the agreement between these observations and the mean values for Ca/P ratio, as observed in previous experiments, in the same bone sites and animals. Collagen fibril diameter and Ca/P ratio can both serve as indexes of bone quality.

## INTRODUCTION

In bone, collagen represents more than 90% of the organic bone matrix. It confers resistance to the structure and establishes the biomechanical properties of the tissue[1]. The collagen fibril diameter has been regarded as the most important factor related to biomechanical strength of tissues[2,3,4]. To understand the biomechanical strength of various bone sites, therefore, it is important to study this structural parameter of collagen fibrils in these sites.

Regarding the bone mineral content, it is generally accepted that its measurement is an appropriate estimate of bone strength. Changes in the amounts of Ca and P in biological apatites do not go hand in hand; therefore, a decrease in bone density may be due to a decrease in either Ca or P, or to dissimilar decreases in both. Consequently, the determination of the Ca/P ratio may provide a sensitive measure of bone mineral changes and may add to our understanding of the changes occurring in bone diseases.

It is known that the mechanical strength of bone depends, first of all, on the condition of the cortical bone[5,6]. In this work, the effect of bone site and sex have been included by using cortical femoral neck, front and rear tibia samples from female and male rats. Comparisons between these results with those derived from a different experimental animal, rabbit, have also been included. The aims of this study were to:

1. Compare the mean collagen fibril diameter values between bone sites, genders, and animal species
2. Use the collagen fibril diameter as step towards understanding its importance as an indicator of bone strength
3. Compare the mean collagen fibril diameter values with the previously obtained[7,8,9] bulk Ca/P ratio values as indexes of bone quality

## MATERIALS AND METHODS

### Animals — Bone Samples

Female and male Wistar rats and rabbits, 18 and 7 months of age, respectively, were used. Animals were bred and housed in natural conditions and killed under light ether anesthesia. Throughout the experiments, care was taken to minimize pain or discomfort. All studies were approved by the Ioannina University Institutional Animal Care and Use Committee.

Cortical front and rear tibia, as well as femoral neck, samples from 20 normal experimental animals, 10 Wistar rats, and 10 New Zealand albino rabbits, were analyzed. Half of each group of species was females and half males. The mean weight of the rats was 292 ± 10.6 g with a range from 280–300 g, while that of the rabbits was 2.46 ± 0.21 kg with a range from 2.2–2.7 kg.

### Preparation of Specimens for Electron Microscopy

Samples of each chosen bone site were taken from the right side. They were carefully freed from soft tissue, and prepared for electron microscopy. The tissue was fixed in a solution containing 4 ml of 0.05 *M* phosphate buffer (pH 7.2), 0.5 ml of 25% glutaraldehyde, and 0.5 ml of 12.5% ethylene-diamine tetraacetic acid (EDTA) disodium salt. After 2 h, the bone was administered changes of 2 ml of EDTA in 3 ml of phosphate buffer until decalcified. Bone samples were trimmed into approximately $0.5mm^3$ cubes and fixed in 2.5% glutaraldehyde in 0.05 *M* phosphate buffer (pH 7.2). Specimens were washed in the same buffer before and after fixation and rinsed in distilled water. Fixed specimens were then dehydrated in a graded alcohol series and impregnated overnight with 1:1 mixture of propylene oxide, and the resin used for embedding. Final embedding was performed in capsules with agar resin. Polymerization was completed in 48 h at $60^oC$.

Ultrathin sections were positively stained with 2% aqueous solution of phosphotungstic acid (PTA; pH 3.3) for 1 h and then with 2% aqueous solution of uranyl acetate (UA; pH 4.3) for 30 min, washing briefly in distilled water before and after UA staining.

### Electron Microscopy

Electron microscopy was performed on a JEOL JEM 100 CX-II electron microscope and micrographs were taken at ×8,000–20,000. Grating replicas were used for magnification calibration.

### Morphometric Measurements

For measurements of the diameter of collagen fibrils, areas of cross-sectional collagen were photographed. A minimum of 400 collagen fibrils from at least 4 micrographs were analyzed for each subject. Thus, as in each case 5 animals were involved, the diameters of at least 2,000 fibrils from 20 micrographs were averaged for each bone site. Measurements were made on prints from electron micrographs by the use of an algorithm developed in the laboratory. First the image of each electron micrograph, i.e., cross sections, was transferred by a GT 6000 Scanner to digital form and stored into the computer. This transfer was made using the program P-Styler with a resolution of at least 480 dpi. The digital image was converted into binary, through thresholding, by the program pixel.exe. A vertical and horizontal scanning of the final image was performed, in order to measure the dimensions and structural characteristics of each region. Fibril diameter is measured in pixels, which is subsequently converted to nm using the dpi of the transferred image. Further details appear in Tzaphlidou and Berillis[10].

Statistical analysis was performed according to Snedecor and Cochran[11] and Hays[12]. The mean and standard deviation, as well as data from an unpaired *t*-test, were calculated. The significance of the difference between the two samples compared was estimated by using a *p* value following the statistical methods described by Snedecor and Cochran[11].

## RESULTS

Table 1, in the first column of figures, summarizes the mean diameter values (with standard deviation) of female rat collagen fibrils from various cortical bone sites. There were significant differences ($p < 0.001$) in mean values between the femoral and front tibia, as well as between the rear and front tibia. A significant difference ($p < 0.004$) was also detected between the femoral and rear tibia. A comparison has been made between these values and those obtained from the age-matched, male rats[13]. In these subjects, statistically significant differences ($0.001 < p < 0.04$) were detected between different bone sites, i.e., between femoral and front tibia, femoral and rear tibia, as well as rear and front tibia. In both genders, the greatest mean diameter value is that for the femoral and the smallest for the front tibia.

**TABLE 1**
**Mean Diameter Values of Rat Collagen Fibrils from Various Bone Sites**

| Bone Sites | Mean Diameter Values and Standard Deviation (nm) | |
|---|---|---|
| | Female | Male[13] |
| Front tibia | 34.5 ± 7.1 | 47.1 ± 10.4 |
| Rear tibia | 38.1 ± 6.5 | 48.8 ± 12.2 |
| Femoral | 39.5 ± 6.5 | 50.4 ± 11.8 |

The results are also similar between females and males when collagen fibrils are from rabbit’s various bone sites. The first column of figures in Table 2 shows the mean diameter values (and standard deviation) obtained from female rabbits. The examined bone sites are the same with those in rats. These values are compared with those from the age-matched male animals shown in the right-hand column of the table. The results, in both genders, point to a significant difference in mean value ($p < 0.001$) between front and rear tibia, as well as between rear tibia and femoral. No statistically significant difference was detected between front tibia and femoral. It is worth noting that in rabbits, in both genders, the greatest mean diameter value is that for the rear tibia and the smallest for the front tibia.

## DISCUSSION

The samples processed for electron microscopy showed statistically significant differences in the collagen fibril diameter between different bone sites in most cases studied. The samples were made up of collagen fibrils from different genders and species.

**TABLE 2**
**Mean Diameter Values of Rabbit Collagen Fibrils from Various Bone Sites**

| Bone Sites | Mean Diameter Values and Standard Deviation (nm) | |
|---|---|---|
| | Female | Male |
| Front tibia | 42.7 ± 9.1 | 48.3 ± 5.4 |
| Rear tibia | 46.3 ± 8.7 | 53.2 ± 6.9 |
| Femoral | 43.1 ± 9.9 | 49.3 ± 7.2 |

The order of magnitude for mean diameter value is the same for females and males in both kinds of experimental animals. As it is shown in Tables 1 and 2, there are statistically significant differences in the collagen fibril diameter between female and male subjects. As has been reported[14], this is not related to bone site, but to sex. It is worth noting that no statistically significant differences in mean collagen fibril diameter values between cortical and trabecular bones either from rats[15] or rabbits[16] have been detected. While in rats the greatest mean diameter value is that for the femoral, the greatest mean value in rabbits is that for the rear tibia. In addition, in all cases the smallest mean value is that for the front tibia. Different life activities arising from the evolutionary adaptation of these species could be considered as a possible explanation for the observed difference. Rats have been adapted for running, while rabbits for jumping. These movement activities exert differing pressure conditions to their front and rear legs and, as a consequence, at least their bone strength has to be respectively adapted. According to this postulation, in rats the femoral neck should have better strength than the other bone sites, while in rabbits, the rear tibia should be the strongest one.

Similar variations in the mineral part (mean Ca/P ratios) of the above bone sites using the same kind of experimental animals have also been demonstrated[7,8,9]. By using synchrotron microCT, these authors reported significant differences in mean Ca/P values between different bone sites; in addition, while in rats, the mean Ca/P value for cortical femoral is significantly greater than that of front and rear tibia, in rabbits, the greatest mean value is that for the rear tibia, demonstrating a dependence on lifestyle and bone use. Furthermore, the smallest mean Ca/P value for both species is that for the front tibia, which is in agreement with the smallest value for collagen fibril diameter.

It appears that a relationship exists between collagen fibril diameter and the Ca/P ratio for the same kind of experimental animal and bone site. Both of these parameters can be used as indexes of bone quality[17]. Mechanical bone strength depends on the mass and geometry of bone and the structural (architectural and micro architectural), or quality, parameters of bone tissue. As has been reported[18,19,20], the Ca/P ratio may provide high reliability for the diagnosis, prevention, and treatment of bone disorders. In addition, alterations in the skeletal Ca/P ratio with disease may be indicative of underlying changes in the organic matrix of bone, mainly collagen[17]. Also, lines of evidence suggest that there is a relationship between osteoporosis and alterations in the bone Ca/P ratio[7,17,21] and in bone collagen fibril diameter[15,16]. As bone Ca/P ratio and collagen fibril diameter decline in parallel with osteoporosis, the present observations may have a significant input in understanding the pathogenesis of some of the most troublesome and painful bone disorders that afflict mankind.

## ACKNOWLEDGMENTS

PB has been partially supported by grant no. 62/1405 of Ioannina University Research Committee.
